# Comparison of treatment planning for stereotactic radiosurgery and stereotactic body radiation therapy techniques with 2.5 mm and 5 mm multileaf collimator (MLC): A pilot study

**DOI:** 10.12688/f1000research.141178.1

**Published:** 2024-04-18

**Authors:** Rechal Nisha Dsouza, Krishna Sharan, Suresh Sukumar, Srinidhi G Chandraguthi, Shreekripa Rao, Shirley Lewis, Umesh V, Senthil Manikandan

**Affiliations:** 1 Medical Radiation Physics Program, Manipal College of Health Professions, Manipal Academy of Higher Education, Manipal, Karnataka, 576104, India; 2 Department of Radiotherapy and Oncology, K S Hegde Medical Academy, Mangalore, Karnataka, 575022, India; 3 Department of Medical Imaging Technology, Manipal College of Health Professions Manipal, Manipal Academy of Higher Education, Manipal, Karnataka, 576104, India; 4 Department of Radiotherapy and Oncology, Kasturba Medical College of Manipal, Manipal Academy of Higher Education, Manipal, Karnataka, 576104, India; 5 Department of Radiation Physics, Kidwai Memorial Institute of Oncology, Bengaluru, Karnataka, 560029, India

**Keywords:** Stereotactic Radiotherapy, Stereotactic Body Radiotherapy, micro multileaf collimator(mMLC), Multi leaf collimator (MLC), Treatment planning, Volumetric Modulated Arc Therapy, Dynamic Conformal Arc Therapy

## Abstract

**Background:**

The Elekta Apex micro-multileaf collimator (mMLC) for planning stereotactic radiosurgery (SRS)/stereotactic body radiation therapy (SBRT) provides excellent dose distribution; however, it offers disadvantages such as prolonged treatment duration and technical errors in terms of mMLC and gantry calibration, which adds to the total treatment duration. Hence, we aimed to compare the treatment planning performed with the 2.5 mm Apex mMLC and 5 mm MLC (Elekta Agility) for brain and lung targets treated with SRS and SBRT in Elekta Versa high definition (HD).

**Methods:**

The study included 10 patients, five each with brain and lung targets. Two treatment plans were performed for each case using Elekta’s Monaco (5.11.03) treatment planning system (TPS) with 2.5 mm and 5 mm MLC. An X-ray photon beam of energy 6FFF was used for planning purposes with various gantry, couch, and collimator combinations. These two plans were compared using target coverage (TC), conformity index (CI), homogeneity index (HI), gradient index (GI), and organ at risk (OAR) doses.

**Results:**

No significant differences were found in the target coverage, CI, HI, or OAR doses in either MLC design. Volumetric modulated arc therapy (VMAT) with a 5 mm MLC provided equivalent tumor coverage with an additional number of monitor units. OAR doses were comparable in both MLC widths for brain targets, whereas for lung targets, OAR doses were slightly lower with 2.5 mm mMLC. GI was superior in the 2.5 mm mMLC compared to the 5 mm MLC giving a steep falloff in the dose distributions (p = 0.158).

**Conclusions:**

The TC, CI, HI, and OAR doses were similar in both 2.5 and 5 mm based VMAT plans. The gradient index was better in the 2.5 mm mMLC resulting in steep dose gradients, which further reduced the isodose volumes. Therefore, a 5 mm MLC (agility) can also be used for SRS/SBRT treatment planning, with a further reduction in the gradient index. However, the study must be extended further with more samples and multiple comparison parameters.

Clinical Trials Registry - India, registration number CTRI/2021/11/037842, registration date. 8 ^th^ November, 2021.

## Introduction

Cancer is considered one of the leading causes of death worldwide, and its incidence and mortality rates may rise to approximately 29 million and 16 million people, respectively, by 2040. ^1^ In India, the incidence rate of brain or nervous system-related cancers is the leading cause of death and is ranked 13th in males and 11th in females among all cancers. ^1^ Radiotherapy is an extremely cost-effective treatment modality, and almost 50% of cancer patients receive radiation therapy during their course of cancer treatment. ^2^ In radiotherapy, high-energy photons such as X-rays, gamma radiation, or high-energy particles are used to deliver treatment externally using external beam radiation therapy (EBRT), or they can be administered internally using radioactive sources such as brachytherapy. ^3^

Stereotactic radiosurgery (SRS) is a radiotherapy treatment technique in which a high radiation dose is delivered in a single fraction, using multiple radiation beams or arcs. If the same treatment is delivered in multiple fractions, it is called stereotactic radiotherapy (SRT). ^4^ For selected intracranial lesions, SRS/SRT is performed as it delivers a high amount of radiation to the tumor volume, and the dose gradient is steep so that the surrounding uninvolved structures are spared. ^5^ Lars Leksell, a Swedish neurosurgeon, initially introduced SRS in 1969 for treating intracranial lesions and has successfully shown positive results in managing intracranial lesions and brain metastasis. It has increased the tumor control rate, survival rate, and quality of life. An SRS system based on a linear accelerator (LINAC) was developed in 1980, which allowed the treatment of SRS, SRT, and stereotactic body radiation therapy (SBRT) with high precision and accuracy. ^6^ Similar to SRS, SBRT is an SRT procedure that is used to treat extracranial lesions where hypofractionated radiation is administered in a fewer number of fractions. SBRT has been regularly incorporated as a better treatment option for metastatic and primary tumors such as lung cancer, liver lesions, and prostate cancer. ^7^

SBRT is one of the best therapeutic options for inoperable liver and other metastases of the body. Patients with primary and metastatic liver lesions treated with 46–50 Gy in four–six fractions have shown better local tumor control as well as overall survival. ^8^ For brain tumors less than 3 cm, treatment with SRS given in a single fraction has shown the best results as it has various advantages such as a greater decrease in tumor size, sparing of normal tissues due to rapid dose fall-off, minimum radiation-related side effects, lesser duration of radiotherapy, and better tumor response. If the tumor size is greater than 3 cm, SRS must be delivered in multiple fractions (SRT) so that normal tissue toxicity is minimized. ^9^

SRS/SRT/SBRT are complicated treatment procedures that include various steps such as immobilization of the patient, patient imaging, delineation of the target (tumor) and critical structures, treatment planning, performing patient-specific quality assurance (QA) checks, and delivering radiation therapy to the patient, including patient follow-up. ^10^ As high radiation doses are delivered in a smaller fraction of treatments, very small error margins are given compared to conventional radiation therapy. A small error in the localization of the tumor, even in a single fraction, can lead to an underdosage of the tumor by 20% or more, and at the same time, it can overdose the surrounding normal tissues, thereby resulting in potentially serious injuries to the organs involved. ^11^

The treatment delivery of stereotaxy has generally utilized dedicated specialized equipment such as Gamma Knife and CyberKnife. Medical LINACs have been increasingly adopted as a mode for delivering hypofractionated radiotherapy schedules. There are important advantages to using a LINAC for SRS/SRT/SBRT treatment, including increased versatility, cost benefits, and higher throughput. Technological advances have made use of LINAC and are increasingly accurate in stereotaxic treatment. One such device, designed to improve conformity to the target, is an externally attached additional collimating device with an mMLC. ^12^ The mMLC had a high-resolution collimation system with a width of less than 3 mm at the isocenter. This would facilitate the delivery of a conformal radiation dose to the tumor with a steep dose gradient beyond the tumor, thus better sparing the surrounding normal tissues. However, there is a disadvantage of reduced clearance between the patient and collimator, which limits the utilization of non-coplanar beam directions. ^13^

Using the Elekta Apex mMLC for planning SRS/SRT/SBRT provides excellent dose distribution, but also offers technical errors in terms of mMLC and gantry calibration, which adds to the total treatment duration. If the same plan with excellent output is available without the use of Apex mMLC but with flattening filter free (FFF) photon beam energies, then the treatment duration is expected to be reduced compared to Apex-based delivery.

This study aimed to compare SRS/SRT/SBRT treatment plans with and without the mMLC, using FFF radiation beams to compare the generated treatment plans in terms of various indices of planning target volume (PTV) coverage and organ-at-risk doses.

## Methods

### Patient selection

Ten patients were retrospectively considered for the study after approval from the Institutional Ethics Committee, Kasturba Medical College and Kasturba Hospital, Manipal Academy of Higher Education Manipal (IEC427-2021) 8 ^th^ August 2021, Clinical Trials Registry, India; registration number CTRI/2021/11/037842, 8 ^th^ November 2021. population included five patients each with brain and lung targets who were treated in the year 2021 at our institute. The eligibility criteria for the patient selection was based on the guidelines given. ^14^^–^^17^ The selected cases were planned using SRS and SBRT treatment techniques following the guidelines prescribed by the Radiation Therapy Oncology Group (RTOG) and Stereotactic Ablative Body Radiotherapy (SABR) guidelines using the Monaco 5.11.03 (Elekta, 2016) treatment planning system (TPS). For every patient, two plans were generated with the Apex mMLC (2.5 mm) and the other without the mMLC (i.e., with the Elekta Agility, 5 mm). The gantry, collimator, and couch angles were determined based on the tumor location and kept constant in both treatment plans.

### Patient immobilization for SRS

The selected patients were immobilized with a Fraxion patient-specific cranial immobilization device (Fraxion, P10106-103) consisting of a computed tomography (CT) adaptor, tabletop adaptor, vacuum cushion, thermoplastic mask, and Fraxion stereotactic frame. Vacuum cushions from Fraxion are specially used as head rests for patients, and they provide precise positioning of the head and reproducibility of the treatment setup. Each cushion was molded individually for each patient and used throughout the course of treatment. ^18^ Each patient was immobilized using a thermoplastic mask. A CT scan of the patient was performed with the Fraxion stereotactic frame and marking sheet. A stereotactic frame was used to locate the tumor in SRS/SRT. It has three Z-shaped radiopaque markers that are visible in the axial cut CT image as nine dots that act as fiducials for the identification of the target coordinates. The marking sheet has three coordinates, that is, x, y, and z, which are used to locate the tumor in the coordinate system. This makes it convenient to position patients according to the treatment isocenter. The acquired CT images were exported to the Monaco TPS. Magnetic resonance imaging (MRI) is considered superior to CT in soft-tissue discrimination of the brain. Image registration was performed, and mapping of the structures was carried out. Contouring of the MRI image onto the CT image was performed for the exact delineation of the target and critical structures such as the normal brain, optic nerves, optic chiasma, brainstem, eyes, lens, cochlea etc. ^19^

### Immobilization for SBRT

Selected patients were immobilized using a thermoplastic mask. An active breathing coordinator (ABC) (Elekta limited, 201510) was used to implement effective respiratory motion management throughout the treatment. ^20^ An ABC is used to control respiratory motion by applying simple and efficient breath-holds at an applicable threshold level. A CT image of the patient was obtained with the same setup using a thermoplastic mask and ABC. ^21^ Image fusion using MRI and CT was performed to properly delineate the tumor volume and critical structure, where appropriate.

### Contouring and treatment planning

A CT image with a slice thickness of 1 mm for the brain and 2 mm for the lungs was acquired using suitable immobilization devices. For SBRT cases, CT images with deep inspiration breath-hold (DIBH) were acquired. ^22^ The CT images were then exported to Monaco (5.11.03 version) TPS. ^23^ Contouring of the target volume and organ at risk (OAR) was accomplished by oncologists, and a later treatment plan was planned based on the site of the tumor using Monaco TPS. ^24^ For this study, two separate treatment plans for SRS and SBRT were generated retrospectively.

**Figure 1.**
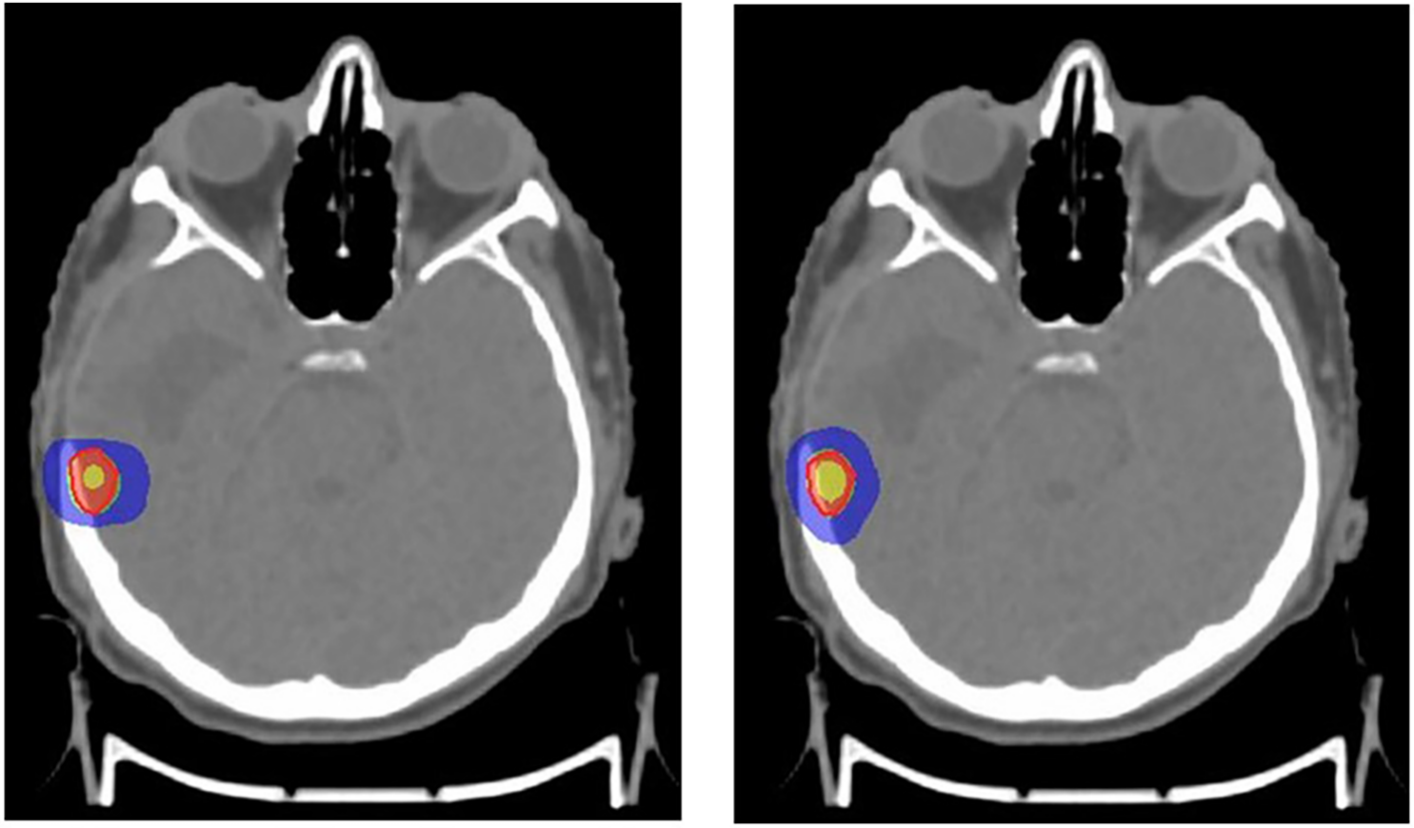
Stereotactic radiosurgery (SRS) plan with APEX 2.5 mm micro-multileaf collimator (mMLC) (left), SRS plan with Agility 5 mm multileaf collimator (MLC) (right). Red: 100% prescribed dose; Green: 95% prescribed dose; Blue: 50% prescribed dose; Yellow: 115% prescribed dose.

**Figure 2.**
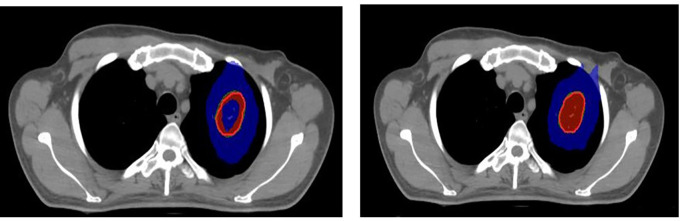
Stereotactic body radiation therapy (SBRT) plan with APEX 2.5 mm micro-multileaf collimator (mMLC) (left), SBRT plan with Agility 5 mm multileaf collimator (MLC) (right). Red: 100% prescribed dose; Green: 95% prescribed dose; Blue: 50% prescribed dose; Blue (inside): 115% prescribed dose.

The first plan for SRS/SBRT was developed using the dynamic conformal arc therapy (DCAT) technique with a 6FFF photon beam energy using a 2.5 mm Apex mMLC. The Apex mMLC is an additional attachment to the collimator of the Elekta Versa high-definition LINAC consisting of an MLC with a 2.5 mm width at the isocenter. This high-resolution collimating device is especially used for SRS treatment delivery to facilitate conformal dose distribution around the tumor. The maximum field size provided by the mMLC is 12 × 14 cm ^2^. ^25^ The isocenter was placed at the center of the target volume. The number and direction of the arcs were chosen based on the location of the tumor. Couch movement was restricted in some cases, wherever it was practically impossible to move the gantry with the Apex to reduce the risk of collision of the gantry with the couch and patient. The second plan was generated without using Apex mMLC. The LINAC has an inbuilt MLC with a 5 mm width at the isocenter, which is also called Agility. The SRS/SBRT treatment plans in this case were performed with agility using a 6FFF photon beam energy. The treatment plan was implemented using the same gantry couch combinations. Multiple optimizations in both techniques were performed to achieve the prescribed tumor dose and to bring the dose to the OARs within the given limits. The Monte Carlo algorithm was used to calculate the dose distribution. ^26^ For the included cases, SRS was planned with 16 Gy in one fraction ( Figure 1) and SBRT with 60 Gy in five fractions ( Figure 2).

### Plan evaluation

The treatment plans performed using APEX mMLC and agility were compared using Monaco TPS. The quality of the treatment plans was checked using quality indices such as target coverage (TC), conformity index (CI), homogeneity index (HI), gradient index (GI), and organ at risk (OAR) doses. CI was calculated using the formula TV _PIV_^2^/ (TV × PIV). Here, TV is the target volume, and PIV is the prescription isodose volume. GI was calculated using the formula PV _50%_/PIV. PV _50%_ represents 50% of the prescribed dose covered by the patient volume. The HI was calculated as the ratio of the maximum target dose to the prescribed dose. ^27^ The ideal value for HI and CI is 1, and as it decreases from 1, the quality of the plan also decreases. A value greater than 1 indicates that the tumor volume is overirradiated, and a value less than 1 indicates a reduction in the dose to the target volume. The smaller the GI value, the steeper the dose gradient. If multiple targets are close to each other, then combined GI will be performed for the lowest dose prescribed target in the patient. A clinically acceptable plan will have a lower GI value, higher CI value, and higher TC (>95%). Such a plan will provide better tumor coverage and maximum sparing of the normal brain.

### Statistical analysis

Jamovi 2.3.26 ^28^ statistical software was used for statistical analysis and descriptive data for all continuous variables were presented as mean ± standard deviation (SD). Multivariate ANOVA and independent t-test were performed to compare the treatment plans generated with 5 mm and 2.5 mm MLC for brain SRS and lung SBRT. A value of p<0.05 was considered as a statistically significant difference between variables.

## Results

**Figure 3.**
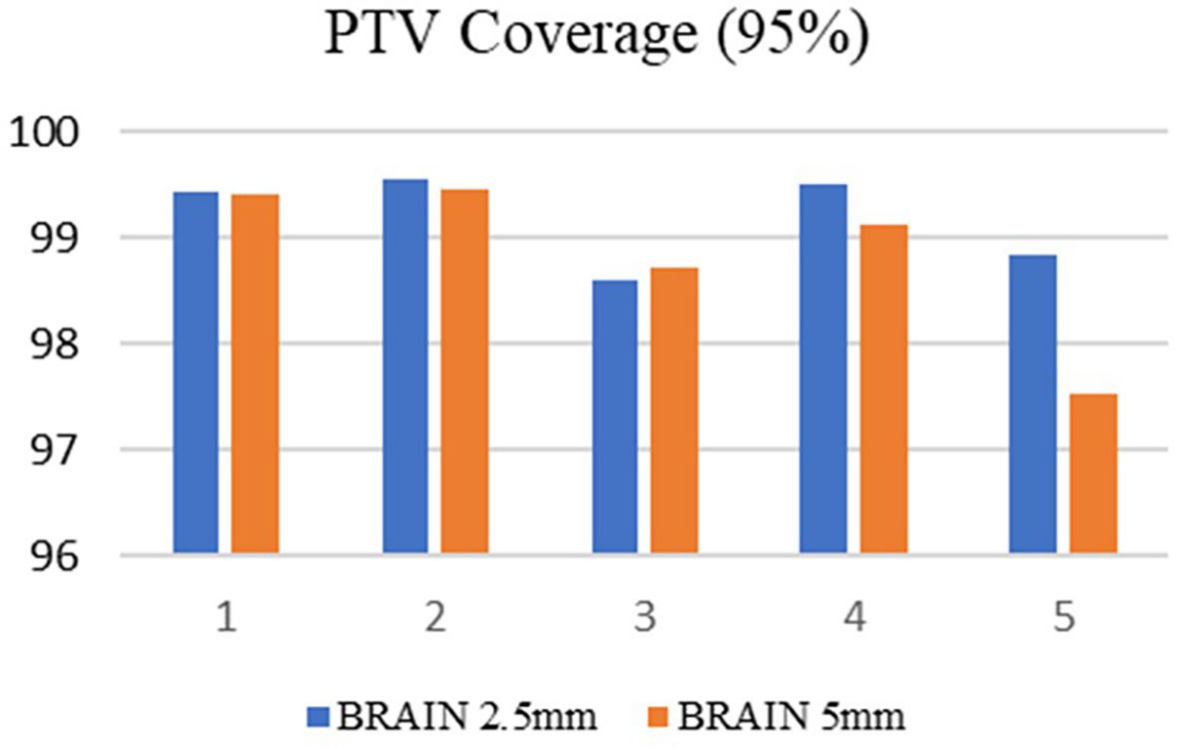
Planning target volume (PTV) coverage in the brain with 2.5 mm and 5 mm.

**Figure 4.**
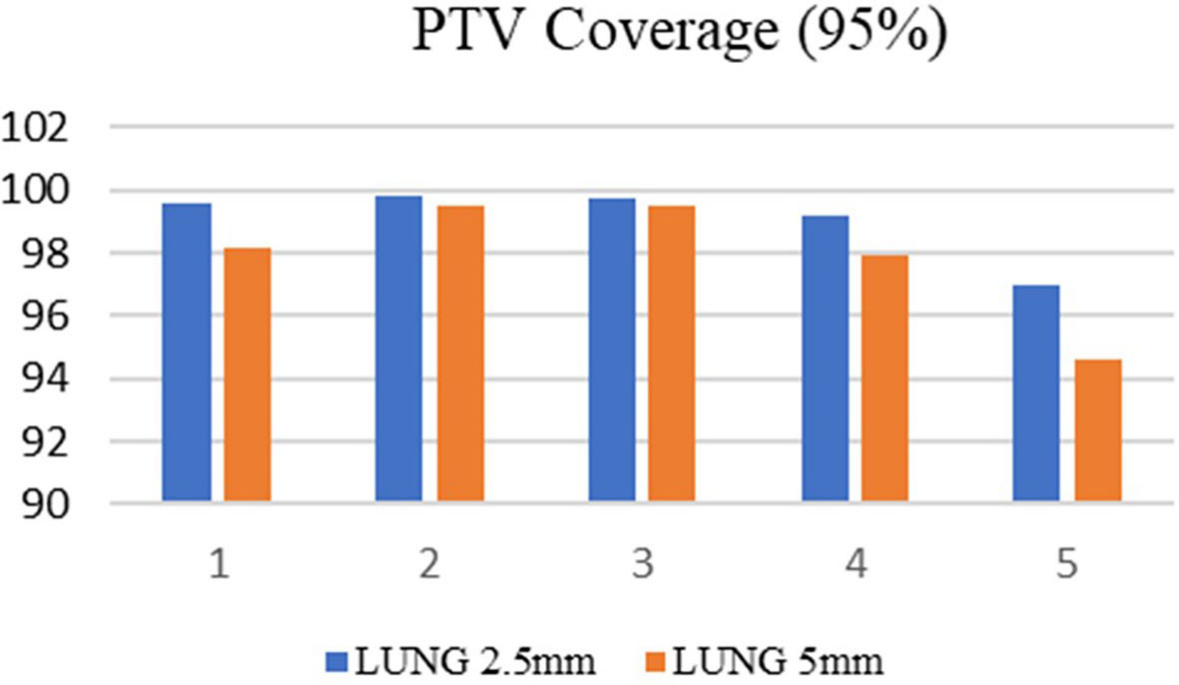
Planning target volume (PTV) coverage in the lung with 2.5 mm and 5 mm.

The volume of the GTV and PTV in the selected brain targets were ranging between 0.6 cm ^3^ to 6.0 cm ^3^ and 1.2 cm ^3^ to 8.5 cm ^3^ respectively. Similarly, in the lung targets the GTV and PTV volumes were ranging between 4.3 cm ^3^ to 7 cm ^3^ and 8.5 cm ^3^ to 12.5 cm ^3^ respectively. Among the selected ten cases, 50% of the patients were treated at the metastatic sites, and in the remaining cases, treatments were administered at the primary sites. Both treatment planning techniques showed excellent tumor dose distribution and the PTV coverage was comparable in both treatment planning techniques. For brain SRS cases, the mean target coverage in agility (5 mm MLC) was 98.85±0.78692 and in APEX (2.5 mm) it was 99.196±0.43592(p=0.415) ( Figure 3). In lung SBRT cancers, the target coverage in Agility and APEX was 97.934±2.02090 and 99.076±1.17980(p=0.307) respectively ( Figure 4) which shows that the APEX-based treatment plans showed slightly better target coverage, although both planning techniques provided a target coverage of more than 97%.

**Figure 5.**
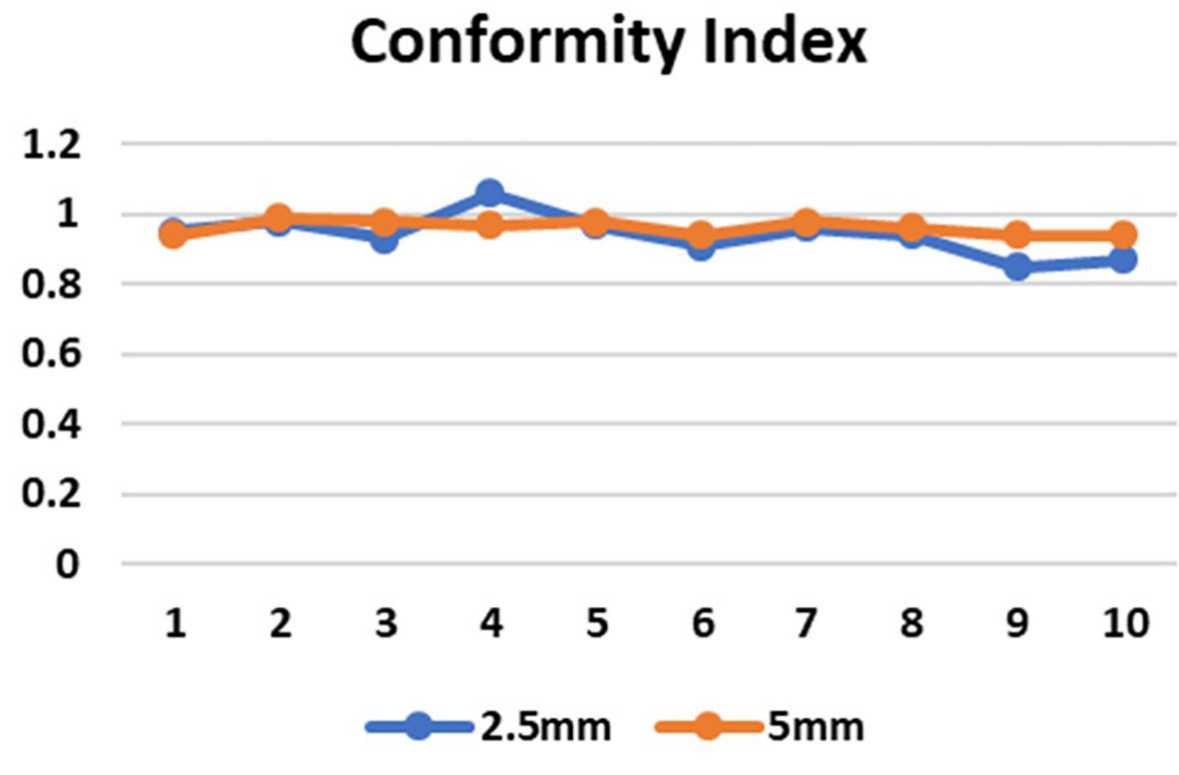
Conformity index (CI) in 2.5 mm and 5 mm multileaf collimator (MLC) for 10 cases.

**Figure 6.**
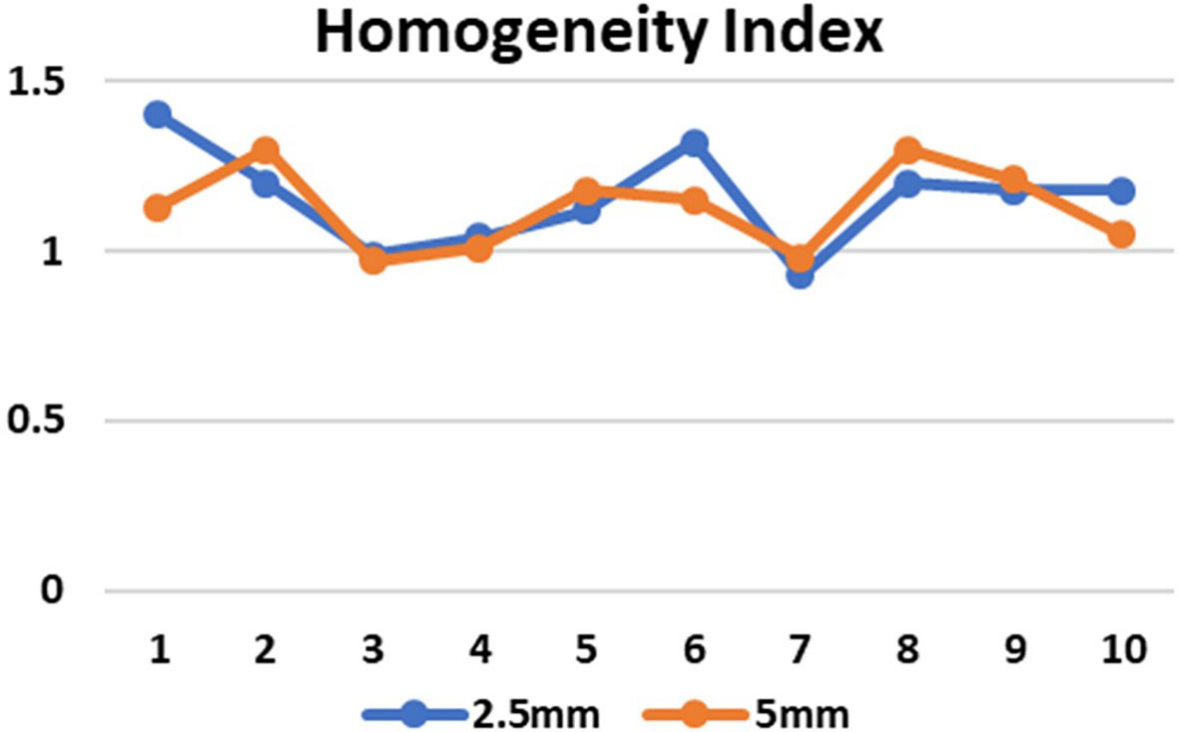
Conformity index (CI) in 2.5 mm and 5 mm multileaf collimator (MLC) for 10 cases.

**Figure 7.**
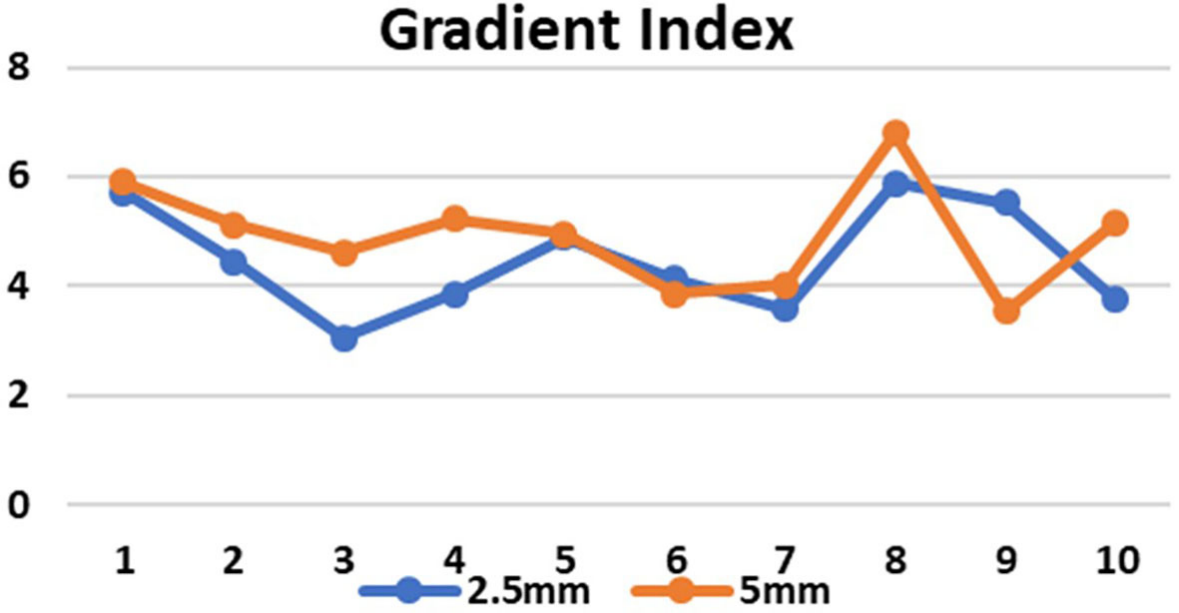
Gradient index (GI) in 2.5 mm and 5 mm multileaf collimator (MLC) for 10 cases.

CI in both the SRS and SBRT plans yielded excellent results. These values were found to be similar for both the planning techniques. In the 5 mm and 2.5 mm width MLC the mean CI for the brain targets were 0.972±0.01924 and 0.9780±0.04970 respectively (p=0.808) and for the lung targets were 0.9520±0.01789 and 0.9060±0.04615 respectively (p=0.071) which indicates that the target area is optimally covered ( Figure 5) and there is no significant difference in the CI for brain and lung targets in both MLC designs. The HI values in case of 2.5 mm and 5 mm MLC in brain targets were 1.150±0.16093 and 1.1180±0.13293 respectively (p=0.741) and for the lung targets the values were 1.1620±0.14220 and 1.1380±0.12677 respectively (p=0.785). The HI values were also found to be close to unity, indicating a homogenous dose distribution across the tumor volume ( Figure 6). The GI values for 5 mm and 2.5 mm MLC in brain targets were 5.1660±0.47732 and 4.3880±1.00912, respectively (p=0.158), and for the lung targets 4.670±1.33318 and 4.5720±1.05611, respectively(p=0.895). Although there was no significant difference between the GI values, the obtained results showed that GI was better in the 2.5 mm APEX based plans compared to 5 mm MLC ( Figure 7). The mean and maximum doses to the OARs in both treatment planning techniques for both 2.5 mm and 5 mm MLC based plans were found to be close to each other. Although there was no significant difference between the two, it has been shown that the doses were slightly lower in most of the OARs in lung targets, whereas in brain targets, we received mixed results, indicating that both techniques are comparable ( Tables 1 and 2). Data on the same have been made available online. ^29^

**Table 1.** Organ at risk (OAR) doses in stereotactic radiosurgery (SRS) brain with 5 mm and 2.5 mm multileaf collimator (MLC).

Brain targets			
Organ dose (Gy)	5 mm MLC	2.5 mm MLC	p-value
Brainstem (max)	3.842±2.63541	3.006±1.80263	0.574
Whole Brain-PTV (mean)	0.712±0.29626	0.61±0.31369	0.611
Cochlea Left (mean)	0.874±0.72834	1.17±1.22417	0.655
Cochlea Right (mean)	0.792±0.72479	0.66±0.35143	0.724
Eye Left (mean)	0.1794±0.206063	0.252±0.224544	0.609
Eye Right (mean)	0.284±0.23298	0.308±0.30178	0.892
Lens Left (mean)	0.17±0.12268	0.244±0.20169	0.503
Lens Right (mean)	0.438±0.23868	0.536±0.29416	0.579
Optic Chiasma (max)	1.058±0.61145	0.9±0.66208	0.705
Optic Nerve Left (max)	0.688±0.56305	0.488±0.25685	0.490
Optic Nerve Right (max)	1.026±0.47479	1.086±0.55851	0.859

**Table 2.** Organ at risk (OAR) doses in stereotactic body radiation therapy (SBRT) lung with 5 mm and 2.5 mm multileaf collimator (MLC).

Lung targets			
Organ dose (Gy)	5 mm MLC	2.5 mm MLC	p-value
Spinal Cord (max)	14.606±1.821841	12.448±2.347266	0.069
Heart (mean)	1.028±0.543926	0.834±0.60042	0.154
Lung-PTV (mean)	4.47±0.723657	3.994±0.654021	0.042
Right Lung (mean)	1.142±0.16754	1.08±0.22439	0.634
Left Lung (mean)	5.504±0.54866	5.362±0.53607	0.348
Bronchus (mean)	16.57±1.49970	15.338±2.28067	0.342
Esophagus (mean)	2.566±0.50659	2.452±0.82612	0.799

## Discussion

Almost 10–30% of all carcinoma patients have a risk of brain metastasis, which can originate from primary sites such as the lung, renal cell cancer, breast cancer, colorectal cancer, and melanoma. ^30^ Multiple options are available for the treatment of brain metastasis, including radiation therapy to the whole brain, surgical resection of the tumor, SRS, and fSRT. SRS is considered the first-line treatment for tumors that can be destroyed without invasive techniques. SRS is recommended if the patient has a minimum number of sites of metastasis in the brain and is preferably used for patients with a good overall physical condition. SRS and fSRT have been shown to have potential benefits in terms of good tumor control and reduced toxicity. ^31^ SRS administered in single and multiple fractions (fSRT) has provided good local control, with a lower risk of radiation-related necrosis. SRS can be used if the diameter of the tumor is less than 3 cm, and SRT in two to five fractions is basically used for larger lesions or when surgical removal of the tumor is not advisable. Therefore, the ultimate goal of SRT is the reduction of adverse effects due to the large size of the tumor and the improvement of local control. ^32^

The lung is one of the most common sites of metastasis in various primary regions. On average, approximately 20–54% of cancer patients will suffer from lung metastasis in their lifetime. ^22^ Radiotherapy administered in multiple fractions plays an important role in terms of overall survival and high tumor control rates compared to other palliative care, including treatment cost, which is less than or equal to that of surgery. Respiratory management is considered during imaging and treatment to prevent tumor movement. This reduces the toxicity that occurs in relation to the treatment and shoots the target in the exact position in every breathing cycle. Various modalities, such as 4D CT, Active Breathing Coordinator (ABC), and deep inspiration breath-hold (DIBH), Image-guided radiotherapy (IGRT) and cone-beam computed tomography (CBCT)-based treatment delivery help to accurately treat the tumor with very less to no error margin. ^33^ Advanced technology in the LINAC, such as the use of FFF photon beams, lesser width MLC, advanced treatment planning techniques such as Volumetric Modulated Arc Therapy (VMAT), SRS, SBRT, DCAT, and the option of respiratory gating during simulation and treatment, have made SRS/SBRT treatment more optimal and accurate. ^34^

In the current study, ABC was used for SBRT to manage the breathing of patients, and CBCT was used for verification. Accurate treatment was delivered with an error margin of ±1 mm. Contouring of the brain and lung lesions was performed by the physicians after fusing the CT (Philips, 16 slice) images with MRI to draw accurate tumor volume and the OAR. CT is considered the first-line imaging modality used to diagnose the disease and to determine the true extent of the disease. However, the brain and mass in the lung are soft tissues, and the approximate and true spatial extent of the disease cannot be identified using CT images alone. MRI is superior to CT for the identification of soft tissues, definite tumor size, exact tumor location, number of lesions in the brain, and peritumoral edema. Therefore, both CT and MRI images were combined. The actual tumor volume was contoured on MRI and automatically mapped onto the CT image, which was then used for planning purposes. ^32^

This study identified dosimetric differences in the treatment plans performed with the addition of mMLC (2.5 mm) and agility (5 mm). It is important to conform the radiation dose to the target volume in SRS/SRT/SBRT because of the size of the lesion and proximity of the OAR so that the dose distribution is bound tightly to the tumor volume. For this reason, dedicated machines are now available, including standard LINAC with added mMLC. ^35^ However, the addition of this device prolongs the total treatment duration and reduces the clearance between the collimator and patient because of restrictions in choosing the appropriate and required gantry angles most of the time. It has limitations in the placement of noncoplanar arc angles. ^36^ The LINAC (Elekta HD Versa) used in the current study was equipped with an built-in 160 MLC with a width of 5 mm at the isocenter. To deliver treatment plans performed with a 2.5 mm MLC using the same LINAC, an additional collimating device (APEX) must be attached to the machine. APEX consists of 56 pairs of MLC with a 2.5 mm width at the isocenter, which provides a field size of 12 × 14 cm ^2^.

The SRS and SBRT treatment plans performed with a 2.5 mm MLC were considered as the benchmark in this study, and these plans were compared with a 5 mm MLC. The plans with a 2.5 mm MLC were optimized to deliver the best possible treatment with more than 95 to 98% coverage of the target volume with other parameters to be within the prescribed limits. In the plans with a 5 mm MLC, the gantry, couch, and collimator combinations were kept constant, and the treatment plans were optimized to deliver the same dose distribution by keeping all the dose constraints to the OARs minimum. The resulting PTV coverage was equivalent in the 2.5 mm and 5 mm treatment plans. The obtained results were comparable to the results obtained by Wu *et al*., in which three planning techniques were used for the comparison, and the plans were performed using 2.5 mm and 5 mm MLC. They suggested that the PTV coverage, HI, and CI were better in IMRT-based HD MLC plans; however, as the size of the tumor increased and the OARs were closer to the target, the scope of HD MLC also became less noticeable. ^36^

The treatment plan quality was compared using HI, CI, and GI. ^27^ The HI and CI values for both the plans were comparable. Heather *et al*. conducted a study in which they compared MLC of three different widths, 2.5, 5, and 10 mm, for the SBRT spine. With respect to tumor coverage, CI, and HI, the results of the study were comparable irrespective of leaf width. ^37^ Joablot *et al*. conducted a study wherein they performed SRS/SRT plans using 2.5 mm, 5 mm and 10 mm MLC. With respect to HI, they found no clinically significant differences in any of the different MLC widths. In addition, there was no difference in the CI between 5 mm and 2.5 mm for PTV volumes greater than 1cc. However, for PTV volumes lesser than 1cc, the dose conformity was less in the 5 mm MLC, but the amount of reduction was clinically acceptable. The OAR doses were found to be comparable irrespective of leaf width. The dose falloff was steep in the 2.5 mm MLC which was clinically acceptable. ^12^ This result was also observed in the current study in terms of GI, which showed a steep dose fall off in the 2.5 mm MLC. The GI was slightly higher in the 5 mm MLC compared in the 2.5 mm MLC which indicates a steeper dose fall in the 2.5 mm MLC. ^37^ The GI is an important factor that indicates the surrounding normal tissue dose. The lesser the GI, the more the surrounding OARs are spared. ^38^ In a study conducted by Ferrer *et al*., three SRS cases were planned with a 5 mm and 2.5 mm MLC. They obtained similar results for conformation indices and OARs. The target coverage in VMAT is also better at the cost of more monitoring units. They found satisfactory results with VMAT, but in order to have more comparisons between VMAT and APEX, planning must be performed with a larger sample size. ^29^

This study adds to the literature by showing that a 5 mm MLC may suffice in most cases of SRS and SBRT and will be beneficial in reducing the treatment time. This study was limited by the sample size and its retrospective nature.

## Conclusions

There was no clinically significant difference found in the target coverage, CI, HI and OAR doses in ten patients planned for SRS and SBRT using 2.5 mm (APEX mMLC) or 5 mm (agility) MLC with 6FFF photon beam energy. The target coverage was comparable for both the plans. VMAT with agility provided equivalent tumor coverage with an additional number of MUs. The CI and HI values were almost similar in both plans, resulting in a conformal and homogenous dose distribution to the entire PTV. OAR doses were comparable in both MLC for brain cases, whereas for the lung targets, OAR doses were slightly lower with 2.5 mm mMLC. GI was superor in the 2.5 mm mMLC compared to the 5 mm MLC giving a steep dose fall off in the dose distributions. Hence, a 5 mm MLC is also preferred for SRS and SBRT, with GI being an important consideration during planning.

## Data Availability

Harvard Dataverse: Underlying data for ‘Comparison of treatment planning for stereotactic radiosurgery and stereotactic body radiation therapy techniques with 2.5 mm and 5 mm multileaf collimator (MLC): A pilot study’, https://www.doi.org/10.7910/DVN/CFNQEX. ^29^ Harvard Dataverse: STROBE checklist for ‘Comparison of treatment planning for stereotactic radiosurgery and stereotactic body radiation therapy techniques with 2.5 mm and 5 mm multileaf collimator (MLC): A pilot study’, https://www.doi.org/10.7910/DVN/CFNQEX. ^29^ Data are available under the terms of the Creative Commons Zero “No rights reserved’ data waiver (CC0 1.0 Public domain dedication).
