# Supplementary material for: Comparison of treatment planning for stereotactic radiosurgery and stereotactic body radiation therapy techniques with 2.5mm and 5mm multileaf collimator (MLC): A pilot study
Source: F1000Res. 2025 Mar 17;13:290. Originally published 2024 Apr 18. [Version 2] doi: 10.12688/f1000research.141178.2 (PMC11986410; doi:10.12688/f1000research.141178.2)
Supplement: Peer review history

# Reviewer report: Reviewer response for version 2

**DOI:** 10.5256/f1000research.178733.r371419

**Published:** 2025-04-10

**Authors:** Michele Zeverino

**Affiliations:** 1 Institute of Radiation Physics, Lausanne University Hospital and University of Lausanne, Lausanne, Switzerland

No further comments to make.

Is the work clearly and accurately presented and does it cite the current literature?

Partly

If applicable, is the statistical analysis and its interpretation appropriate?

Partly

Are all the source data underlying the results available to ensure full reproducibility?

Yes

Is the study design appropriate and is the work technically sound?

Partly

Are the conclusions drawn adequately supported by the results?

Yes

Are sufficient details of methods and analysis provided to allow replication by others?

Partly

Reviewer Expertise:

I am a Medical Physicist with >15 years of experience in the field of Radiation Oncology. My expertise covers different topics of interest spanning from the implementation of new treatment planning and optimization strategies to the most recent innovations brought by the artificial intteligence in this domain.

I confirm that I have read this submission and believe that I have an appropriate level of expertise to confirm that it is of an acceptable scientific standard.

# Reviewer report: Reviewer response for version 1

**DOI:** 10.5256/f1000research.154595.r281423

**Published:** 2024-09-26

**Authors:** Mary Joan

**Affiliations:** 1 Christian Medical College and Hospital, Punjab, India

The authors have compared SRS and SBRT treatment plans with 2.5 mm and 5 mm MLCs by evaluating target coverage (TC), conformity index (CI), homogeneity index (HI), gradient index (GI), and organ at risk (OAR) doses. The paper is well structured and systematically written. The observations made by the authors add to the existing knowledge and confidence. One drawback with the study is the small number of patients plans recruited for evaluation. As this is a pilot study, the report could be accepted for indexing. The authors are encouraged to continue further with the study.

Is the work clearly and accurately presented and does it cite the current literature?

Yes

If applicable, is the statistical analysis and its interpretation appropriate?

Yes

Are all the source data underlying the results available to ensure full reproducibility?

Yes

Is the study design appropriate and is the work technically sound?

Yes

Are the conclusions drawn adequately supported by the results?

Yes

Are sufficient details of methods and analysis provided to allow replication by others?

Yes

Reviewer Expertise:

Radiological Physics, radiotherapy, treatment planning, dosimetry, radiobiology, quality assurance, research methodology, radiation protection, emergency management

I confirm that I have read this submission and believe that I have an appropriate level of expertise to confirm that it is of an acceptable scientific standard.

# Reviewer report: Reviewer response for version 1

**DOI:** 10.5256/f1000research.154595.r286443

**Published:** 2024-06-05

**Authors:** Michele Zeverino

**Affiliations:** 1 Institute of Radiation Physics, Lausanne University Hospital and University of Lausanne, Lausanne, Switzerland

Introduction

- The first paragraph should also encompass some clinical data regarding the incidence of lung cancers since it is one of the treatments under evaluation.
- I would not mention the use of brachytherapy. Although it is true that it is an option in radiation therapy, I do not see its application in the specific context.
- I would avoid introducing treatment site such as the liver to explain the use of SBRT. Please keep the focus of the paper sticked on the main topic mentioning the use of SBRT for lung cancers instead.
- SRS provides better tumor response with respect to what?
- The various steps listed to describe SRS/SBRT as complicated procedures actually apply also to fractionated EBRT. Please be more specific.
- The authors stated, “As high radiation doses are delivered in a smaller fraction of treatments, very small error margins are given compared to conventional radiation therapy”. Well, the margins for SRS/SBRT are smaller because of the use of high precision machines and/or the use of immobilization devices. Please reformulate this sentence, accordingly.
- Spell out the word mMLC as it was firstly introduced in the manuscript.
- Why did the authors write that the leaf width of the mMLC is less than 3mm instead of reporting its real measure (2.5mm)?
- Which are the technical errors and gantry calibration issues the authors refer to? If this is a limitation of the system that lead them to justify the use of the 5mm MLC instead, then more arguments are needed to support the aim of the study.
- The vendors name and country should be cited in brackets after each medical device listed in the Introduction section.

Methods

 Patient selection

- Which RTOG? And what are the SABRT guidelines used? Please include at least the references.

Patient immobilization for SRS

- Which kind of MRI was used? I also suggest reporting that rigid image registration was carry out between MRI and CT to avoid any misinterpretation.

Immobilization for SBRT

- Which kind of MRI was used? Was it registered rigidly or not to the CT?

Contouring and treatment planning

- I suggest moving the first paragraph in the previous sections as it looks more appropriate to define slice thickness and structure delineation in the description of imaging protocol.
- Was the dose prescribed to the same isolines for both rival plans. This is a very important element to report within a planning comparison study! And in the specific case of SRS, SBRT this may have a serious impact on the results! Looking at Figure 1, I have the feeling that this was not the case (i.e. same dose but different isolines prescriptions).
- Please report the PTV margins used and to which structure they were applied (CTV or GTV).

Plan evaluation

- I would recommend to authors using throughout the text the same definition for the mMLC and MLC plans to avoid confusion.
- Here there is a problem concerning the HI definition. In SRS/SBRT, the dose is typically prescribed to the 80% or smaller isolines to increase the steep dose fall-off outside of the PTV. Therefore, HI will always be larger than unity as reported in the results. The sentence concerning the over/underdosage of the target with respect to values larger/smaller than unity applies only to CI and not to both HI and CI.

Statistical analysis

- Due to the small sample size, the use of a t-test looks not appropriate. Did the authors check the normality of their distributions? If not, I would recommend using a non-parametric test instead to assess any statistical difference.

Results

- Please avoid the term “excellent” as these results are aligned with a typical treatment planning evaluation protocol for SRS/SBRT.
- I would recommend using only one decimal digit for mean and SD target coverage (by the way add also the unit of measure (%)). The same suggestion applies also for HI, CI, and GI.
- Why in Figures 5,6, and 7 the results are reported with no distinctions between brain and lung cases?
- I would also add the resulting MU comparison, or if they were very similar between plans this should be reported at least in the Discussion section.

Discussion

- I found the three initial paragraphs of the Discussion section useless as they are not focused on the discussion of the results but rather, they look like a repetition of the Introduction section.
- What do the authors mean when they claim, “Accurate treatment was delivered with an error margin of ±1 mm”? Was it the setup error of the delivery system or what else? Please be more specific.
- I believe that a fairer comparison should have also included targets located more adjacent to OARs. In that case, mMLC is expected to outperform MLC. As this was not done in the study, please comment on that in the Discussion section.

Is the work clearly and accurately presented and does it cite the current literature?

Partly

If applicable, is the statistical analysis and its interpretation appropriate?

Partly

Are all the source data underlying the results available to ensure full reproducibility?

Yes

Is the study design appropriate and is the work technically sound?

Partly

Are the conclusions drawn adequately supported by the results?

Yes

Are sufficient details of methods and analysis provided to allow replication by others?

Partly

Reviewer Expertise:

I am a Medical Physicist with >15 years of experience in the field of Radiation Oncology. My expertise covers different topics of interest spanning from the implementation of new treatment planning and optimization strategies to the most recent innovations brought by the artificial intteligence in this domain.

I confirm that I have read this submission and believe that I have an appropriate level of expertise to state that I do not consider it to be of an acceptable scientific standard, for reasons outlined above.
